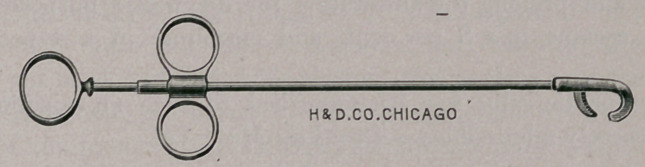# Department of Canine, Feline, and Avian Medicine and Surgery

**Published:** 1902-05

**Authors:** Cecil French

**Affiliations:** D.V.S. (McGill and Munich), Washington, D. C.


### DEPARTMENT OF CANINE, FELINE, AND AVIAN MEDICINE AND SURGERY.

By Cecil French, D.V.S. (McGill and Munich),
WASHINGTON, D. C.

A Condition of Neoplasia Exhibiting Progressive Malignancy. Of late years pathologists have recognized the fact that certain forms of tumor which in the first instance exhibit innocent characteristics may later undergo progressive transformation into a condition of malignancy. This is particularly true of the class of epithelial or granular growths approximating to fully formed tissue, of which adenomata form a noteworthy example. It will be remembered that an adenoma is an innocent growth originating from pre-existing glandular tissue and formed by proliferated gland cells arranged in an orderly manner and supported by a fibrous stroma. But the cells differ from normal ones in that they have no power of producing the normal secretion peculiar to the gland tissue from which they grow. That is to say, if any secretion at all is produced, it is a modified one, and the gland has no means of discharging it externally by proper ducts. There are adenomas of the liver which clearly show bile pigmentation, and thyroid adenomas may lead sometimes to exophthalmic goitre, brought about by excessive production of excretion, which often disappears upon removal of the tumors. Tumors of the class under consideration show no tendency to infiltration of the neighboring lymphatics, but it is certain that they may undergo progressive transformation into true malignant carcinomatous structures. So long as the overgrowth of follicles tends to retain the glandular form it is termed an adenoma, but as soon as the overgrowth is characterized by infiltration and development of an embryonic type of cells it is termed a carcinoma.

One of the first veterinarians to observe this phenomenon was Leblanc, in the year 1858 (*Rec. de Med. Veter.*, p. 911). He made consecutive examinations of recurring mammary tumors, and observed a gradual transformation into malignancy. The first growth removed was found to be a simple adenoma, but malignant characteristics became more and more accentuated as the recurrence increased in frequency. In an instance recorded by M'Fadyean, a carcinoma appeared to have developed as a secondary growth from an anal adenoma. In another instance which I observed, a firm mammary tumor appeared in a female Skye terrier, at the age of eight years. After reaching a moderate size it remained quiescent for seven years, when it suddenly commenced to enlarge at an alarming rate, in addition to giving birth to numerous secondary growths in the neighboring glands. It proved on examination to be a carcinoma.

The case which forms the substance of this report is a very interesting one. The animal in question is a male mongrel hound, aged about eight years. For some years it has been secured by a chain, one end of which was attached to a ring which ran on a wire about forty feet long. In this manner the animal was enabled to run up and down within a limited area. On its right side, as it left its sleeping quarters, was a high wall, while on the other side, a short distance away, was its master's residence. Hence, on running up and down the length of the wire, it would invariably get on the side looking toward the house, and it so happened that the chain tended always to get between its front legs and rub continuously on a certain spot on the posterior and inner aspect of the right leg. About two years ago an abrasion was noticed on this spot, and this gradually gave place to a sessile fibrous growth which grew slowly for eighteen months. It then suddenly began to increase in size at a much more rapid rate, its weight causing it to become pediculate.

Recently I removed this growth and forwarded it to Professor Adami, of the McGill Pathological Laboratory, for histologic diagnosis. In his report, Professor Adami states that the main mass is fibromatous, but here and there included in it are certain little areas which, to the naked eye, look paler and yellower, and when squeezed give out a fatty substance. Under the microscope similar areas show these to be apparently sebaceous glands undergoing what, under careful examination, could not be regarded as otherwise than an early cancerous change. That is to say, that the masses of cells become more irregular and can be traced passing into the surrounding stroma. Therefore the diagnosis *is fibro-adenoma with early malignancy.*

This case also demonstrates once more what has been so often noticed, viz., that the final result of chronic irritation of a part may be a malignant neoplasm, and that it is always advisable to observe the rule of early and free removal of neoplasms whenever they are in accessible situations.

A NEW CANINE AND FELINE INSTRUMENT. The accompanying figure illustrates the model of an instrument which Haussmann & Dunn have constructed at my suggestion. It is termed an oöphorectome, and, as the name indicates, is intended for castrating the bitch. It works on the principle of the emasculator, but in the direction of the long axis of the shaft, and this feature, together with the smallness of the blades, which are just large enough to grasp the tissues at either extremity of the ovary after the broad ligament has been torn, enables the operator to sever the tissues at the proximal extremity with facility and to dispense with ligaturing.

The instrument is equally adapted to castration of the male cat.

## Figures and Tables

**Figure f1:**
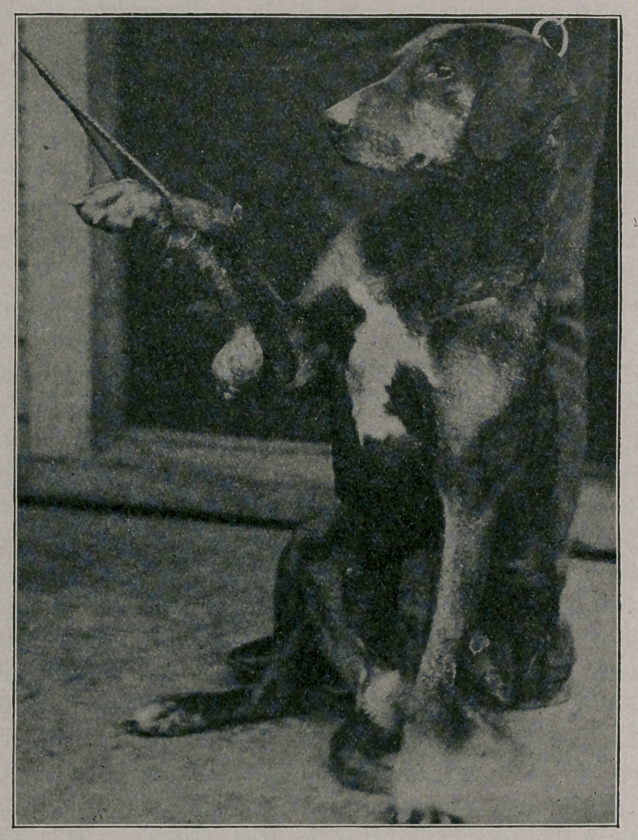


**Figure f2:**